# Analytical evaluation of three soluble transferrin receptor measurement systems for diagnosis of iron deficiency anemia: A retrospective study

**DOI:** 10.1002/jcla.23342

**Published:** 2020-04-22

**Authors:** Li'an Hou, Jun Lu, Xianyong Jiang, Xiuzhi Guo, Chaochao Ma, Xinqi Cheng

**Affiliations:** ^1^ Department of Laboratory Medicine Peking Union Medical College Hospital, CAMS Beijing China; ^2^ Department of Hematology Peking Union Medical College Hospital, CAMS Beijing China

**Keywords:** anemia of chronic disease, chronic iron deficiency anemia, iron deficiency anemia, soluble transferrin receptor

## Abstract

**Background:**

Soluble transferrin receptor (sTfR) is a promising indicator of iron deficiency anemia (IDA). Here, we investigated the application value of sTfR assays based on three different methods for the diagnosis of IDA.

**Methods:**

The sTfR concentrations in two groups of patient specimens with high‐level and low‐level sTfR concentrations and in quality control materials were measured four times a day for five consecutive days to evaluate the precision of the three methods. We selected patients with IDA, anemia of chronic disease (ACD), or chronic diseases with iron deficiency anemia (CIDA), and apparently healthy subjects, and measured the serum sTfR concentrations in all subjects using the three different methods. The cutoff points for an IDA diagnosis using the three assays and their corresponding clinical sensitivities and specificities were calculated by receiver operating characteristic analysis.

**Results:**

For the diagnosis of IDA, the cutoff points of sTfR measured by the chemiluminescent, immunoturbidimetric, and immunonephelometric assays were 2.91, 6.70, and 2.48 mg/L, respectively. The corresponding sensitivities were 85.59%, 85.59%, and 85.59%, the specificities were 91.47%, 90.31%, and 90.70%, and area under the curve was 0.943, 0.944, and 0.936, respectively. The sTfR concentrations measured by the different methods were significantly higher in the IDA and CIDA groups than in the other two groups (*P* < .05).

**Conclusions:**

The sTfR based on the three different measurement methods presented promising analytical performances and met the clinical requirements for sensitivity and specificity. However, the different measurement methods had markedly different cutoff points for IDA diagnosis, which should be critically considered in clinical practice.

## 1 INTRODUCTION

Anemia is a global public health problem. The World Health Organization (WHO) reported that approximately 30% of the world's population suffered from anemia, with children and pregnant women being majorly affected.^1^, ^2^ Microcytic hypochromic anemia is a common type of anemia, and iron deficiency anemia (IDA) is the most common clinical type of microcytic hypochromic anemia. IDA occurs widely globally, especially in developing countries. In the World Health Report 2002 by WHO, IDA was considered one of the top ten causes of the global burden of disease, with an yearly increase of IDA among preschool children and pregnant women.^3^

The current gold standard for an IDA diagnosis is iron staining of a bone marrow smear. However, bone marrow puncture is an invasive measurement method that is cumbersome to perform. In addition, this method is greatly affected by the physical condition and compliance of the patient and is not suitable for large‐scale screening of IDA. Other laboratory indicators for iron deficiency mainly include serum iron (SI), transferrin (TRF), transferrin saturation (TS), and the total iron‐binding capacity (TIBC). SI is greatly affected by physiological and pathological factors,^1^, ^4^ and no significant changes are found in SI, TRF, TS, and TIBC at the storage iron depletion stage in early IDA.^5^ Therefore, these indicators cannot be used individually for the diagnosis of early IDA. Serum ferritin (SF) has a good correlation with storage iron and exhibits high sensitivity. However, no definite diagnosis of IDA can be made with SF in the range of 20‐100 μg/L.^6^, ^7^ In addition, SF, which is an acute phase reaction protein, may increase or remain at normal levels in diseases such as inflammation and infection and does not truly reflect the amount of storage iron. Thus, SF cannot be used to diagnose concomitant IDA in patients with chronic diseases^5^ ^.^

Transferrin receptor (TfR) is a transmembrane glycoprotein.^8^ Iron is transported by binding to specific TfR‐transferrin complex and thereby released into cells.^9^ Through proteolysis, TfR produces soluble transferrin receptor (sTfR) in the serum, whose concentration is proportional to the TfR concentration.^10^ sTfR is mainly derived from early erythrocytes in the bone marrow and can accurately reflect the TfR level on the surface of erythroid hematopoietic precursor cells. The level starts to increase before a significant decrease in the hemoglobin concentration is observed,^11^, ^12^ reflecting the whole process from iron storage depletion to IDA.^13^ When IDA occurs, the serum sTfR concentration is elevated to accelerate the transfer of iron into cells; the erythropoietin (EPO) level is also elevated to increase erythroid hematopoiesis. Therefore, during IDA, an increase in the sTfR level appears first, followed by a decrease in SI and an increase in TIBC^14^ ^.^ sTfR is regulated by the intracellular iron concentration and is not affected by inflammation, infection, or malignancy. Thus, sTfR is a potential indicator for determining whether a patient with chronic disease has concomitant IDA.

Currently, the methods used for sTfR measurement are enzyme‐linked immunosorbent assay (ELISA), chemiluminescent assay, immunofluorescent assay, and immunonephelometric assay. Different measurement results for sTfR are obtained with different reagents and methods, and the reference intervals also vary. Therefore, understanding the exact differences between various methods and the cutoff points for IDA diagnosis using each method is critical for the clinical diagnosis and identification of IDA. The main objectives of this study were to evaluate the analytical performance of three sTfR measurement methods (namely chemiluminescent, immunoturbidimetric, and immunonephelometric assays) and to investigate the differences between the three measurement methods. The cutoff points for IDA diagnosis of the different methods and their corresponding clinical sensitivities and specificities were also determined.

## 2 MATERIALS AND METHODS

### 2.1 Subjects individuals

In this study, we enrolled 436 subjects in Peking Union Medical College Hospital from March 2014 to August 2015. Among these, 118 were patients with IDA, 161 were patients with anemia of chronic disease (ACD), 60 were patients with chronic diseases with iron deficiency anemia (CIDA), and 97 were apparently healthy subjects (HS).

The inclusion criteria^15^ were as follows: (a) IDA patients: red blood cells (RBCs) < 4.0 × 10^12^/L in both men and women; hemoglobin (Hb) < 120 g/L in men and <110 g/L in women; mean corpuscular volume (MCV) < 80 fl; mean corpuscular hemoglobin (MCH) < 27 pg; and mean corpuscular hemoglobin concentration (MCHC) < 0.32. Iron staining of bone marrow smears showed that the stainable iron disappeared from the bone marrow particles, with <15% sideroblasts; (b) ACD patients: RBCs < 4.0 × 10^12^/L in men and <3.5 × 10^12^/L in women; Hb < 120 g/L in men and <110 g/L in women; and hematocrit value (HCT) < 0.35 in patients with diabetes, chronic renal insufficiency, tumor, infection, rheumatoid arthritis, or liver cirrhosis. Iron staining of bone marrow smears revealed no iron deficiency; (c) CIDA patients: RBCs < 4.0 × 10^12^/L in men and <3.5 × 10^12^/L in women; Hb < 120 g/L in men and <110 g/L in women; and HCT < 0.35 in patients with diabetes, chronic renal insufficiency, tumor, infection, rheumatoid arthritis, and cirrhosis. Iron staining of bone marrow smears showed that the stainable iron disappeared from the bone marrow particles, with < 15% sideroblasts; (d) HS group: Subjects older than 18 years old who underwent a physical examination and had normal indicators for complete blood counts and hepatorenal functions.

All of the samples were collected from clinical residual serum. Samples with hemolysis, jaundice, or lipemia were excluded. To avoid the influence of factors such as the stability of reagents and instruments, sera were separated from all specimens and stored in a –80°C freezer prior to uniform measurement. This study was approved by the Ethics Committee of Peking Union Medical College Hospital.

### 2.2 Instruments and reagents

Beckman Coulter DXI800 automatic immunoassay analyzer (Beckman Coulter), Roche Cobas c702 automatic biochemistry analyzer (Roche), and Siemens BNII special protein analyzer (Siemens) with their corresponding sTfR reagents and calibrators were used. The three analytic systems were designated A, B, and C, respectively. All instruments were in good condition and were used normally. The calibration, quality control, and operation procedures were performed according to the manufacturers' instructions.

### 2.3 Method precision

Two different sTfR concentrations of pooled serum and two different sTfR concentrations of commercialization quality control were prepared for precision verification (Bio‐rad). All of the pooled serum and quality controls were aliquoted and stored at −80°C until analysis. Precision samples were measured four times a day for five consecutive days according to Clinical and Laboratory Standards Institute EP 15‐A.^16^ Repeatability and within‐laboratory coefficient verification% (CV%) were calculated.

### 2.4 Method comparison

The sTfR content in the samples was detected by three systems, A, B, and C, and the consistency of the results obtained by the three methods was analyzed.

### 2.5 Clinical application

The cutoff values of the three methods for IDA diagnosis were calculated, and the diagnostic specificity and sensitivity of the different methods were compared.

### 2.6 Statistical analysis

Data analysis and preparation of graphs were done using with SPSS 19.0 (SPSS Inc), Medcalc Statistical software 15.0, and GraphPad Prism 6.0. Quantitative results of sTfR are described as the median with quartiles (P25, P75), as abnormal distribution was revealed using D'Agostino test. The sTfR concentrations were compared between the IDA, ACD, CIDA, and healthy control groups using Kruskal–Wallis one‐way ANOVA with *P* < .05 considered statistically significant. The quantitative sTfR results by different measurement methods were compared using Passing‐Bablok regression equations. The regression equation and Spearman correlation coefficient were calculated, and a Bland‐Altman plot was drawn to compare the consistency between two methods. The level of sTfR for three different methods at the maximum Youden index value was taken as optimal cutoff points to diagnose IDA. The corresponding area under the receiver operating characteristic (ROC) curve, clinical specificity, sensitivity, positive predictive value (PPV), and negative predictive value (NPV) were calculated.

## 3 RESULTS

### 3.1 Precision verification

The within‐run and total imprecision of the different methods were verified using the mixed sera from patients and the third‐party quality control materials (Table 1). The imprecision of all three methods was lower than 10%. Among imprecision of the three methods, the CV% for method A was slightly higher than that for the other two methods.

**TABLE 1:** Imprecision of the three sTfR measurement assays

Specimen	Method	Within‐run			Total		
		Mean (mg/L)	SD	CV%	Mean (mg/L)	SD	CV%
S1	A	0.96	0.05	4.2	1.10	0.05	4.9
	B	2.97	0.10	3.3	2.97	0.14	4.8
	C	1.09	0.036	2.3	1.09	0.04	3.5
S2	A	3.75	0.18	4.2	4.29	0.18	4.3
	B	13.47	0.090	0.7	13.48	0.19	1.4
	C	4.38	0.08	1.8	4.38	0.12	2.7
Q1	A	0.55	0.03	4.9	0.63	0.04	5.9
	B	1.45	0.07	4.5	1.46	0.11	6.3
	C	0.54	0.01	2.5	0.54	0.01	7.9
Q2	A	1.91	0.11	4.8	2.18	0.11	4.8
	B	6.26	0.11	1.7	6.26	0.19	3.1
	C	2.30	0.06	2.7	2.30	0.08	3.4

Note

S1 and S2 are two different sTfR concentrations of pooled serum, and Q1 and Q2 are two different commercial sTfR concentrations for quality control. Beckman Coulter DXI800 automatic immunoassay analyzer, Roche Cobas c702 automatic biochemistry analyzer, and Siemens BNII special protein analyzer with their corresponding sTfR reagents and calibrators were designated A, B, and C, respectively.

### 3.2 Method comparison

**FIGURE 1:**
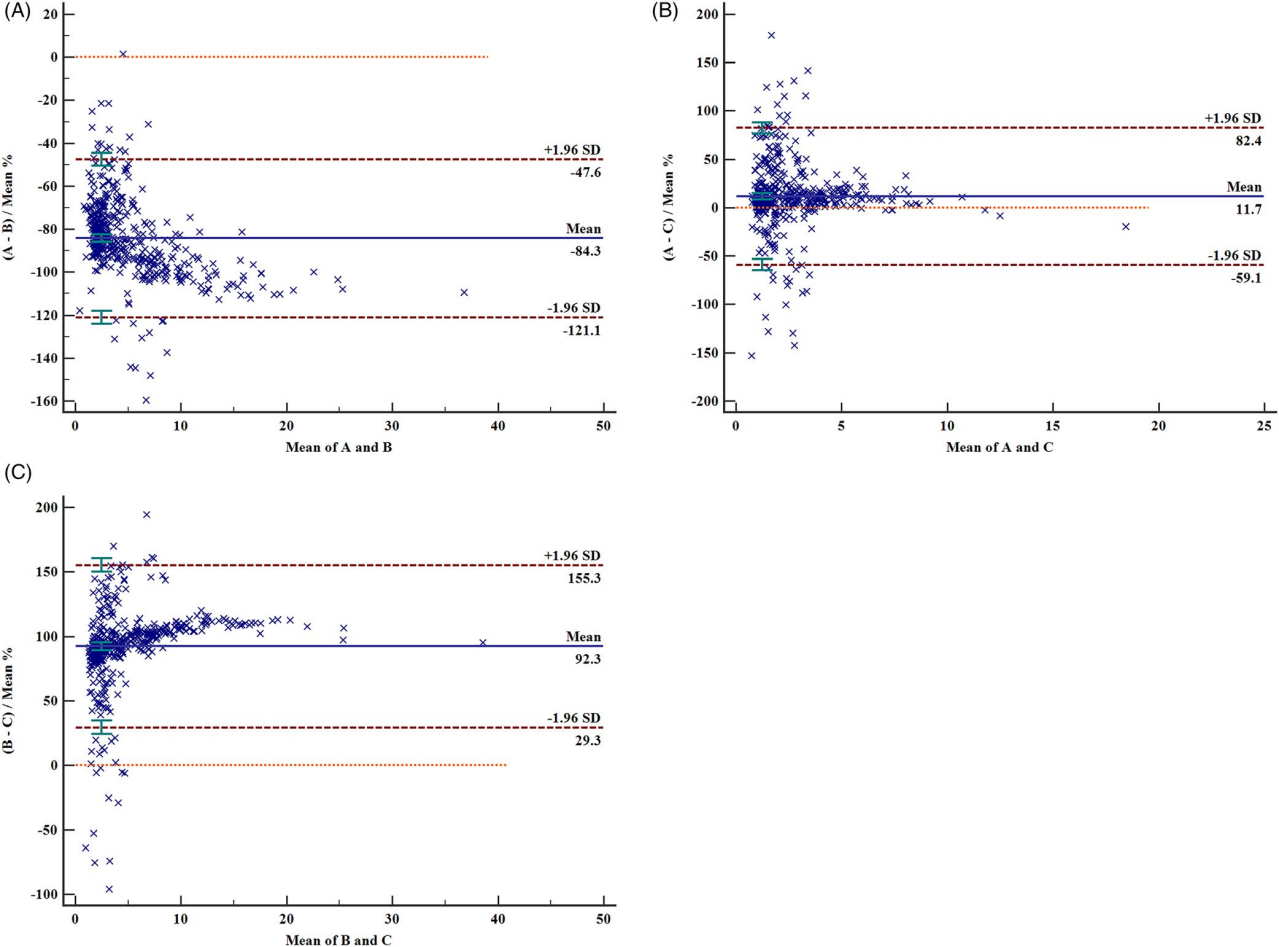
Inter‐assay agreement (A, B, and C) by Bland‐Altman plot. Beckman Coulter DXI800 automatic immunoassay analyzer, Roche Cobas c702 automatic biochemistry analyzer, and Siemens BNII special protein analyzer with their corresponding sTfR reagents and calibrators were designated A, B, and C, respectively. In the data distribution fig, the middle line represents the median, and the part between the upper and lower lines is 95% distribution interval

Quantitative comparison of the three sTfR measurement methods indicated that the Spearman correlation coefficient was 0.788 (A vs C, *P* < 00001), 0.799 (B vs C, *P* < .0001), and 0.946 (A vs B, *P* < .0001), respectively. Although these values were more than 0.70, large deviations were observed between the different methods (Figure 1). In Passing‐Bablok regression analysis, 0 and 1 were not included in the 95% confidence interval l (CI) (Table 2) of slope and intercept for three regression equations, except intercept for A vs C.

**TABLE 2:** Passing‐Bablok regression equations comparing assay values

	A vs B	A vs C	B vs C
Slope 95% CI	0.325 (0.317‐0.335)	1.116 (1.085 to 1.148)	3.496 (3.415 to 3.577)
Intercept 95% CI	0.306 (0.267‐0.357)	−0.031 (−0.093 to 0.016)	−1.135 (−1.278 to −0.974)

Note

Beckman Coulter DXI800 automatic immunoassay analyzer, Roche Cobas c702 automatic biochemistry analyzer, and Siemens BNII special protein analyzer with their corresponding sTfR reagents and calibrators were designated A, B, and C, respectively.

### 3.3 Basic characteristic of enrolled subjects

The IDA, ACD, CIDA, and HS groups comprised of 118, 161, 60, and 97 individuals, respectively. The general information and baseline characteristics for these groups are shown in Table 3.

**TABLE 3:** Baseline characteristics of the patients enrolled in the study

	IDA	ACD	CIDA	HS
Number	118	161	60	97
Age (year)	39 ± 12.07	49 ± 18.35	48 ± 18.39	44 ± 13.65
RBC (×10^12^/L)	3.41 ± 0.52	3.32 ± 0.51	3.39 ± 0.47	4.48 ± 0.29
Hb (g/L)	86.18 ± 17.45	99.21 ± 15.44	93.02 ± 14.74	135.68 ± 6.84
HCT (%)	29.00 ± 4.07	29.67 ± 4.37	29.31 ± 3.93	39.79 ± 2.07
MCV (fl)	86.95 ± 18.35	89.95 ± 8.59	87.12 ± 10.17	89.00 ± 3.63
MCHC (g/L)	296.4 ± 36.3	334.7 ± 20.9	316.6 ± 21.7	341.1 ± 7.7
RDW (%)	17.62 ± 2.87	15.47 ± 2.76	17.37 ± 4.15	12.62 ± 0.78
Sideroblasts(%)	11 ± 2.23	30 ± 6.68	11 ± 2.06	‐
sTfR by:				
A (mg/L)	4.13 (3.36,5.81)	1.75 (1.38, 2.51)**^,#^	2.76 (2.07,4.35)*	1.19 (1.06,1.36)**^,#^
B (mg/L)	11.44 (8.91,17.35)	3.94 (3.02,5.76)*^,#^	7.19 (4.67,11.98)**	2.98 (2.62,3.29)**^,#^
C (mg/L)	3.74 (2.80,5.03)	1.46 (1.10,2.17)**^,##^	2.51 (1.68,3.82)*	1.15 (1.01,1.31)**^,#^

Note

RDW was the abbreviation of red blood cell distribution width. Beckman Coulter DXI800 automatic immunoassay analyzer, Roche Cobas c702 automatic biochemistry analyzer, and Siemens BNII special protein analyzer with their corresponding sTfR reagents and calibrators were designated A, B, and C, respectively. Difference between the IDA and other groups: *P < .05 and **P < .01; difference between the CIDA and other groups: ^#^ *P* < .05 and ^##^ *P* < .01.

### 3.4 sTfR content in different disease groups

**FIGURE 2:**
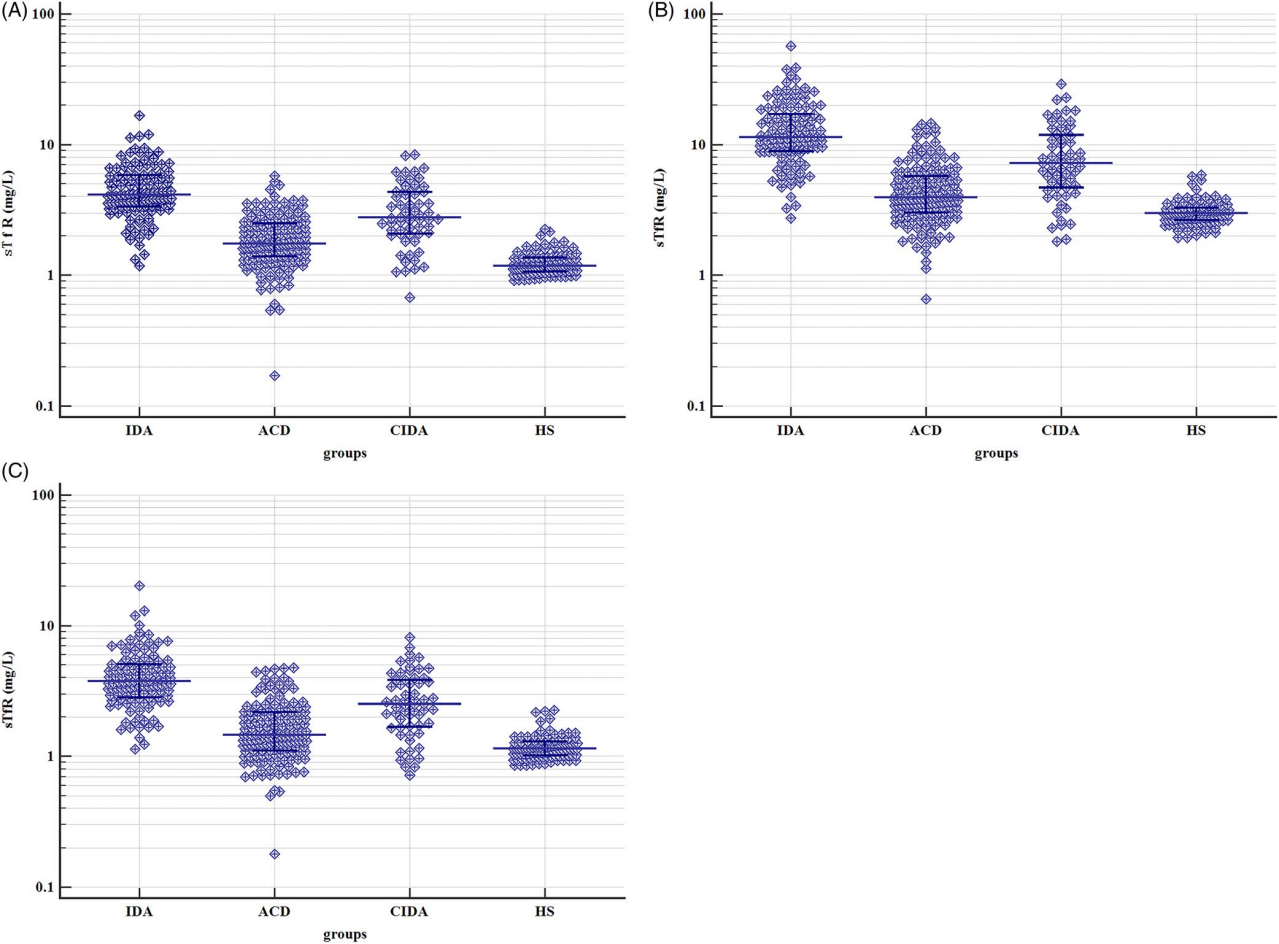
sTfR levels in different groups enrolled in the study measured by the three methods. Beckman Coulter DXI800 automatic immunoassay analyzer, Roche Cobas c702 automatic biochemistry analyzer, and Siemens BNII special protein analyzer with their corresponding sTfR reagents and calibrators were designated A, B, and C, respectively

Quantitative comparison between the different groups revealed that the sTfR concentrations measured by all three methods were significantly higher in the IDA and CIDA groups than in the ACD and HS groups (*P* < .05) (Table 3). Furthermore, significant differences were found when all groups were compared in pairs (*P* < .05). The three methods presented a similar characteristic distribution for sTfR in subjects enrolled in the study; the levels of sTfR in IDA, ACD, and CIDA groups showed a more discrete statistical distribution than that in the HS group (Figure 2). The 95% distribution intervals of the STfR concentrations in the 97 healthy individuals were 0.92‐2.10, 1.97‐5.55, and 0.85‐2.20, respectively.

### 3.5 Clinical application

**FIGURE 3:**
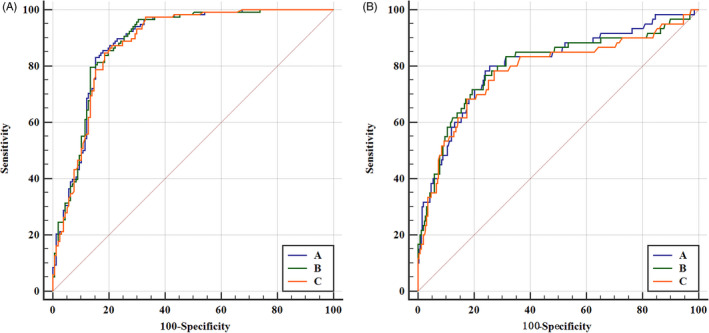
ROC curves for the three measurement methods. Beckman Coulter DXI800 automatic immunoassay analyzer, Roche Cobas c702 automatic biochemistry analyzer, and Siemens BNII special protein analyzer with their corresponding sTfR reagents and calibrators were designated A, B, and C, respectively

The diagnostic efficiency of methods A, B, and C was evaluated by ROC analysis (Figure 3). According to the ROC curves, the AUC for the three methods A, B, and C was 0.943(95% CI: 0.915‐0.964), 0.944 (95% CI:0.9160.965), and 0.936 (95% CI:0.906‐0.959), respectively. The corresponding cutoff points for the diagnosis of iron deficiency were 2.91, 6.70, and 2.48 mg/L, respectively (IDA vs ACD and HS). Based on these cutoff points, the clinical sensitivity, specificity, PPV, NPV, LR(+), and LR(−) were calculated (Table 4). The PPVs of all the three methods were >80%, and their NPVs were >90%. However, the diagnostic efficiency of methods A, B, and C was poor when comparing CIDA patients with ACD patients and HS group. The sensitivity, specificity, PPV, LR(+) in ROC analysis (CIDA vs ACD and HS) were significantly lower than that when comparing IDA patients with ACD and HS groups.

**TABLE 4:** Evaluation of the diagnostic efficiency of the three measurement methods

	Cutoff (mg/L)	Sensitivity (95% CI)	Specificity (95% CI)	PPV (95% CI)	NPV (95% CI)	LR(+) (95% CI)	LR(−) (95% CI)
IDA vs ACD and HS							
A	2.91	85.59% (77.94‐91.38)	91.47% (83.37‐94.58)	82.11% (74.18‐88.44)	93.28% (89.46‐96.04)	10.04 (6.69‐15.07)	0.16 (0.10‐0.24)
B	6.70	85.59% (77.94‐91.38)	90.31% (86.03‐93.63)	80.16% (72.12‐86.73)	93.20% (89.34‐95.99)	8.83 (6.04‐12.91)	0.16 (0.10‐0.25)
C	2.48	85.59% (77.94‐91.38)	90.70% (86.48‐93.95)	80.80% (72.79‐87.29)	93.23% (89.38‐96.01)	9.20 (6.24‐13.56)	0.16 (0.10‐0.25)
CIDA vs ACD and HS							
A	1.95	80.00% (67.67‐89.22)	74.03% (68.23‐79.27)	41.74% (32.61‐51.30)	94.09% (89.90‐96.91)	3.08 (2.42‐3.92)	0.27 (0.16‐0.45)
B	4.62	76.67% (63.96‐86.62)	75.58% (69.87‐80.70)	42.20% (32.80‐52.04)	93.30% (89.02‐96.29)	3.14 (2.43‐4.06)	0.31 (0.19‐0.49)
C	1.63	78.33% (65.80‐87.93)	72.48% (66.60‐77.84)	39.83% (30.93‐49.25)	93.50 (89.14‐96.49)	2.85 (2.24‐3.61)	0.30 (0.18‐0.49)

Note

LR(+): positive likelihood ratio; LR(‐): negative likelihood ratio; NPV: negative prediction value; PPV: Positive prediction value; Beckman Coulter DXI800 automatic immunoassay analyzer, Roche Cobas c702 automatic biochemistry analyzer, and Siemens BNII special protein analyzer with their corresponding sTfR reagents and calibrators were designated A, B, and C, respectively.

## 4 DISCUSSION

Serum sTfR is closely related to the occurrence of iron deficiency in the body. In addition, it is regulated by the intracellular iron concentration, without being affected by inflammation, infection, or malignancy. Therefore, serum sTfR is a promising indicator for determining whether a patient with chronic disease has concomitant IDA. Studies have shown that sTfR can be used for the differential diagnosis of ACD with or without iron deficiency.^1^, ^17^ In the present study, we selected automatic assay kits based on three different methods with relatively high market shares to clarify the precision performance, comparability of measurement results, and efficiency of clinical diagnosis of these kits.

The precision of the three methods was verified as required by the EP15‐A protocol, and all three sTfR assay system showed good precision. The within‐run and total imprecision of the mixed sera were 0.7%‐4.2% and 1.4%‐4.9%, whereas the within‐run and total imprecision of the third‐party quality control materials were 1.7%‐4.9% and 3.1%‐7.9%, respectively. To date, few studies have reported on the methodology used for sTfR measurement. Here, taking the imprecision of the commercially popular sTfR ELISA method (CV% < 10%) as the standard, the imprecision of all three methods tested was found to be lower than that of the ELISA method and thus met the needs of clinical laboratories.

The sTfR results from different disease groups showed that the IDA and CIDA groups had higher sTfR concentrations measured by the three methods than the ACD and HS groups (*P* < .0001). Therefore, the sTfR concentration reflects the condition of iron deficiency in the body and can be used to determine whether an ACD patient has concomitant iron deficiency. In addition, we found that the CIDA group had significantly lower sTfR levels measured by the three methods than the IDA group (*P* < .05). The possible reasons for this finding are as follows^18^, ^19^, ^20^, ^21^, ^22^: TfR expression at the mRNA level is regulated by iron and EPO. When iron deficiency occurs, EPO upregulates TfR expression and thereby increases the sTfR concentration in the serum. However, the inflammatory cytokines produced during chronic diseases may reduce the reactivity of bone marrow to EPO, thereby decreasing the promotion of TfR expression by EPO. Some inflammatory cytokines can also inhibit the proliferation and differentiation of hematopoietic progenitor cells and shorten the lifespan of erythrocytes.^23^ The combined effects of these factors might explain why the sTfR concentration was higher in the CIDA group than in the normal and ACD groups, but lower than that in the IDA group. This effect is also the main reason that sTfR can be used to distinguish between IDA and CIDA. However, in this study, we recruited patients with severe IDA, which may have affected the result. Therefore, we plan to investigate the relationship between sTfR and the severity of IDA in future.

This study showed differences in the numerical results of sTfR measurements among the three different methods. In particular, the measurement results of the immunoturbidimetric assay were significantly higher than those obtained using the other two methods; the average percent deviations were 153.3% and 180.3%, respectively (*P* < .05). In addition, these three different methods had varying cutoff points to diagnose IDA (IDA vs ACD and HS). The cutoff points for sTfR of methods A and C were 2.91 and 2.48 mg/L, respectively, whereas the cutoff point for method B was 6.70 mg/L. The cutoff for method B is significantly higher, which is consistent with the results in previous studies.^24^ This is mainly because of the different traceability of calibrators. The traceability of B has been standardized against an in‐house reference preparation of Roche. In the present study, the 95% distribution interval of the sTfR measurement results in the HS group was slightly higher than the reference interval provided by the manufacturer. This difference may be attributable to the different reference populations selected by the manufacturer; for example, an American population was used for method A, a German population for method B, and a Central European population for method C. The reference intervals of methods A, B, and C given by the manufacturer's instructions were 0.90‐2.01 mg/L (Revised on June 3, 2013); men 2.2‐5.0 mg/L, women 1.9‐4.4 mg/L (Revised on June 18, 2014), and 0.76‐1.76 mg/L (Revised on August 9, 2012), respectively. However, the cutoff points of sTfR determined in this study were all higher than the upper limits of the corresponding reference intervals. Therefore, when using sTfR to evaluate whether a patient has IDA, clinicians should pay particular attention to patients with a sTfR result above the upper limit of the reference interval, but below the cutoff point for an IDA diagnosis. For these patients, the results of other laboratory tests should be considered, and if necessary, iron staining of a bone marrow smear should be performed to confirm the diagnosis. Due to the marked differences between the results obtained with the different methods, different measurement methods should adopt varying reference intervals and diagnostic cutoff points in clinical applications. In addition, the same measurement method should be used in course of treatment monitoring, and the laboratory or measurement method should not be changed optionally. When establishing diagnostic criteria or related guidelines in clinics, attention should also be paid to the differences in the cutoff points of different measurement methods. The detection limit of methods A, B given by the manufacturer's instructions was 0‐12.75 mg/L and 0.5‐40.0 mg/L. The initial detection limit of methods C was 0.14‐4.4 mg/L, and the sample can be diluted 20 times by diluent. Clinical attention should be paid to the ratio of antigen to antibody when using these three methods to detect sTfR. In addition, heterophilic antibodies and M proteins will also affect the detection. At the same time, turbidity and particles in the sample will interfere with the detection results of methods B and C. These are also issues that should be paid attention to.

The clinical specificities of methods A, B, and C were 91.47%, 90.31%, and 90.70%, respectively, all of which were >90% and indicative of a low rate of misdiagnosis. The clinical sensitivities of these three methods were all 85.59%. The AUC values were very close for the three methods (0.943, 0.944, and 0.936, respectively). Therefore, the different methods are generally consistent in the authenticity of their clinical diagnosis. Method A had the highest LR(+), while the LR(−) was similar for the three methods. The diagnostic efficiency of all three methods met clinical needs. However, the diagnostic accuracy when comparing CIDA vs ACD and HS was significantly lower than that when comparing IDA patients vs ACD and HS group. This result is not surprising, because the CIDA group showed significantly lower sTfR levels measured by the three methods than the IDA group. Therefore, measuring sTfR would show better diagnostic accuracy to discriminate between IDA and ACD or HS than that for discriminating between CIDA and ACD or HS. One important limitation of our study is that we did not analyze the factors influencing diagnostic accuracy of sTfR for IDA. Therefore, future studies to discuss the influencing factors are required.

## 5 CONCLUSION

The different sTfR measurement methods showed similar diagnostic value in diagnosing iron deficiency and identifying whether ACD was combined with iron deficiency. However, there were large differences in the measurement results obtained with the different methods, and their cutoff points also varied. Therefore, when sTfR is used in the course of clinical diagnosis and treatment and to establish relevant diagnostic criteria and guidelines, clinicians should pay attention to the differences in the results between different measurement methods. Furthermore, the same measurement method should be used during the course of the treatment monitoring, and optional changes of the laboratory or measurement method should be avoided.
